# Assessing Healthy Aging Score and Its Association With All-Cause Mortality: Findings From the China Health and Retirement Longitudinal Study

**DOI:** 10.1093/geroni/igad006

**Published:** 2023-01-30

**Authors:** Zihang Zeng, Xuerui Li, Wenzhe Yang, Jiao Wang, Yun Zhu, Xiuying Qi, Weili Xu

**Affiliations:** Department of Epidemiology and Biostatistics, School of Public Health, Tianjin Medical University, Tianjin, China; Tianjin Key Laboratory of Environment, Nutrition and Public Health, Tianjin, China; Department of Geriatrics, Tianjin Medical University General Hospital, Tianjin Geriatrics Institute, Tianjin, China; Department of Epidemiology and Biostatistics, School of Public Health, Tianjin Medical University, Tianjin, China; Tianjin Key Laboratory of Environment, Nutrition and Public Health, Tianjin, China; Department of Epidemiology and Biostatistics, School of Public Health, Tianjin Medical University, Tianjin, China; Tianjin Key Laboratory of Environment, Nutrition and Public Health, Tianjin, China; Department of Epidemiology and Biostatistics, School of Public Health, Tianjin Medical University, Tianjin, China; Tianjin Key Laboratory of Environment, Nutrition and Public Health, Tianjin, China; Department of Epidemiology and Biostatistics, School of Public Health, Tianjin Medical University, Tianjin, China; Tianjin Key Laboratory of Environment, Nutrition and Public Health, Tianjin, China; Department of Epidemiology and Biostatistics, School of Public Health, Tianjin Medical University, Tianjin, China; Aging Research Center, Department of Neurobiology, Care Sciences and Society, Karolinska Institutet, Stockholm, Sweden

**Keywords:** Chronic diseases, Cohort study, Environmental support, Healthy aging, Intrinsic capacity

## Abstract

**Background and Objectives:**

To construct a comprehensive healthy aging score (HAS) and explore its association with all-cause mortality and its potential interactions with other demographics on mortality.

**Research Design and Methods:**

This study included 5,409 participants aged ≥60 years from the China Health and Retirement Longitudinal Study. An HAS was constructed based on three dimensions of healthy aging including intrinsic capacity (IC), environmental support (ES), and chronic disease (CD), which were assessed at baseline, and categorized by tertiles (poor, moderate, and high). Participants were followed up biennially for all-cause mortality through the death registration or family interview from 2011 to 2018. Data were analyzed using Cox regression, Laplace regression, and receiver-operating characteristic analysis.

**Results:**

During 7 years of follow-up, 877 (16.21%) participants died. An HAS was constructed based on the cognition, mobility, and instrumental activity of daily living in the IC dimension; housing in the ES dimension; and hypertension, diabetes, chronic lung disease, stroke, and cancer in the CD dimension, which was associated with death. HAS seems a good predictor of all-cause mortality, with an area under the curve of 0.749. The hazard ratios and 95% confidence intervals for all-cause mortality related to moderate and poor HAS (vs high HAS) were 1.26 (1.01–1.56) and 2.38 (1.94–2.91), respectively. The median survival time was 2.46 years shorter in participants with poor HAS than those with high HAS. There were significant additive interactions of HAS with age, sex, and marital status on death.

**Discussion and Implications:**

Poor HAS may increase mortality and shorten survival, especially among older, male, and single adults.

**Translational Significance:** The approach to assess multidimensional healthy aging is warranted. This study constructed a comprehensive healthy aging score (HAS) based on three dimensions of healthy aging including intrinsic capacity, environmental support, and chronic disease, and examined the association of HAS with all-cause mortality, and the interaction of HAS with demographics on mortality among Chinese older adults. These results showed that older adults with poor HAS had higher mortality and shortened survival, especially among older, male, and single adults. Our findings highlight the importance of maintaining intrinsic capacity, improving environmental support, and controlling chronic diseases in healthy aging.

By 2020, the number of people aged 60 years and older has exceeded 1 billion globally. Over the next 30 years, this number is projected to more than double, reaching 2.1 billion (World Health Organization [WHO], 2022). Despite the increasing longevity of older people, the late-life quality among older adults is poor due to diseases and disabilities (Bryant et al., 2019). How to delay the aging process and improve the quality of life to achieve healthy aging is receiving considerable attention (Vogt et al., 2015).

The WHO (2015) has recently redefined the conceptual framework of healthy aging, showing that healthy aging is not limited to identifying a disease-free status. Healthy aging is supposed to be a multidimensional concept that encompasses disease, individual intrinsic capacity (IC, combination of the physical and mental abilities), and extrinsic environmental support (ES, friendliness of the environment), as well as the interaction between them (Davern et al., 2020; WHO, 2015).

The development of a healthy aging indicator for predicting health-related outcomes or mortality has gained interest during the last few years, but there is still a lack of consensus on the assessment of healthy aging. Most studies focused on the IC dimension solely. Among others, clinical biomarkers (e.g., systolic blood pressure, forced vital capacity, and fasting glucose) have been assessed on a scale of 0–2 and summed up to a healthy aging score (HAS), with a higher score indicating unhealthier. These studies indicated that higher HAS was related to higher mortality and risk of disability (Dieteren et al., 2020; O’Connell et al., 2019; Zhang et al., 2021). Other studies focused on the physiological function, integrating different items which indicated physical function, mobility, cognitive function, sensory, and psychology to an HAS, and showed that the HAS was an ideal predictor for death (Camozzato et al., 2014; Daskalopoulou et al., 2019; de la Fuente et al., 2018; Gao et al., 2022; Locquet et al., 2022; Prince et al., 2021). For the ES dimension of healthy aging, previous studies have been limited to sociological factors such as social engagement and social networks (Jaspers et al., 2017; Manierre, 2019; Nosraty et al., 2015). Though the HAS containing these sociological items were observed to be statistically associated with death, the validity of these items was unclear. Besides, the role of surrounding environmental characteristics in healthy aging has been ignored. Several studies explored the association between death and multidimensional healthy aging including disease status, IC-related indicators (i.e., cognitive and physical function), and ES-related indicators (social support and engagement; Kim et al., 2019, 2021; Manierre, 2019; Nosraty et al., 2015; Stickel et al., 2020). However, most of them used a qualitative method to dichotomize healthy aging with yes and no, which was inconsistent with the continuous process of function change (WHO, 2015). Although these studies provide evidence that better performance on the healthy aging dimension is associated with lower mortality, the items used to construct the HAS were empirically motivated and lacked validation (Zamudio-Rodriguez et al., 2021). Moreover, few studies have comprehensively assessed healthy aging from different healthy domains. Open questions remain in the multidimensional construction of healthy aging.

In the present study, we aimed to (a) construct an optimal HAS by selecting the best items in three dimensions associated with death, including IC, ES, and chronic disease (CD); (b) examine the association of HAS with all-cause mortality among older adults; and (c) identify potential interactions of HAS with demographic and lifestyle factors on mortality.

## Method

### Study Population

The China Health and Retirement Longitudinal Study (CHARLS) is an ongoing prospective cohort study that aims to set up a high-quality database representing Chinese households and individuals aged 45 and above. The CHARLS provides a wide range of information from socioeconomic status to health conditions, which fosters interdisciplinary research on aging (Zhao et al., 2014). The baseline survey was conducted between June 2011 and March 2012, and the participants were followed up biennially until 2018 through a face-to-face computer-assisted personal interview (Zhao et al., 2014). More details regarding CHARLS are described elsewhere (Zhao et al., 2014). Among 7,681 participants who were aged ≥60 years, 2,100 with missing data on healthy aging dimensions at baseline and 172 lost during the follow-up were excluded, leaving 5,409 participants for the current study (Supplementary Figure 1).

Informed consent was obtained from all participants. The study was approved by the Institutional Review Board of Peking University. CHARLS data can be requested at http://charls.pku.edu.cn/.

### Data Collection

Data on age, sex, education, marital status, current residence, annual per capita household expenditure, alcohol consumption, smoking status, and healthy aging information (including IC, ES, and CD) were obtained from the questionnaire survey, and participants’ height and weight were measured in the medical examination at baseline.

Education was categorized as no formal education (illiterate), junior high school and below, and senior high school and above. Marital status was grouped into married and single (including separated, divorced, or widowed). The current residence status was dichotomized into rural versus urban. The annual per capita household expenditure was grouped into tertiles (low, moderate, and high). Alcohol consumption was grouped into nondrinking or drinking (including former and current). Smoking status was grouped into never smoking, former smoking, and current smoking. Body mass index (BMI), calculated as weight in kilograms divided by height in meters squared, was classified into underweight (<18.50 kg/m^2^), normal (18.50–23.99 kg/m^2^), overweight (24.00–27.99 kg/m^2^), and obese (≥28.00 kg/m^2^).

Data on death, incident falls, and hospitalization were obtained at the biennial follow-up. Because the exact date of death was not available, the midpoint of the two follow-up visits was defined as the time of death.

### Assessment of Healthy Aging Score

For the assessment of the IC dimension, we used an international scale of healthy aging measurement developed by the Ageing Trajectories of Health—Longitudinal Opportunities and Synergies consortium (Sanchez-Niubo et al., 2021), which was constructed by item response theory approach based on data from 16 international cohorts comprising more than 340,000 individuals. The items included in this scale were in accordance with IC of WHO’s concept (WHO, 2015), covering the biopsychosocial aspects of health and function. In our study, they were harmonized and combined into 30 dichotomous items (presence or absence), covering 7 domains, including cognition (5 items), psychological symptoms (1 item), vitality (3 items), sensory (4 items), mobility (7 items), activity of daily living (ADL, 5 items), and instrumental activity of daily living (IADL, 5 items). A detailed list of items is shown in Supplementary Table 1.

We took a framework of environmental indicators (Davern et al., 2020) as a guide, which was proposed based on WHO’s Age-Friendly Cities and Communities Guide (WHO, 2007) and extensive research evidence, to assess the ES dimension of healthy aging. The following 16 dichotomous items (presence or absence) in 6 domains, including outdoor spaces and buildings (4 items), housing (5 items), communications and information (2 items), community support (3 items), transportation (1 item), and social participation (1 item), were used to measure the ES dimension in the present study. A detailed list of items is shown in Supplementary Table 2.

For the CD dimension, the conditions of chronic noncommunicable diseases were assessed because they were more common in older adults. Fourteen diseases were surveyed by the question “Have you been diagnosed with these diseases by a doctor?”, including hypertension, dyslipidemia, diabetes or high blood sugar, cancer or malignant tumor, chronic lung diseases, liver disease, heart attack, stroke, kidney disease, stomach or other digestive diseases, emotional or psychiatric problems, memory-related disease, arthritis or rheumatism, and asthma. A detailed list of items is shown in Supplementary Table 3.

To construct the HAS, we used “risk score” based on selected variables, which has been commonly utilized in other studies (Gao et al., 2020; Kivipelto et al., 2006). This strategy may decrease the multicollinearity among many variables and ensure practical utilization. Based on a two-step strategy, the outcome-related risk factors were first assessed individually, then those factors that were statistically associated with the outcome were further included in the multivariable regression model. Specifically, in our study, for IC and ES dimensions with two subcomponents (domain and item): (1) univariable analysis was used to examine whether the potential items were significantly associated with mortality, and (2) multivariable Cox regression model was employed to further assess the association between each domain and mortality (summing up corresponding significant items [*p* < .05]). For the CD dimension with only one subcomponent (disease), a two-step process of univariate and multivariate Cox regression analysis was performed to select mortality-related diseases. The scores of IC, ES, and CD dimension were calculated by summing items that were ultimately selected. An HAS was constructed with the equation of 3 × (β1 × IC score + β2 × ES score + β3 × CD score)/(β1 + β2 + β3) according to the previously reported weighted method (Fan et al., 2020), in which the β coefficient, that is, ln(HR), was based on the relationship of IC score, ES score, and CD score (reverse scoring) with death from the Cox regression model. Then an HAS was converted to a *T*-score with a mean of 50 and a standard deviation (*SD*) of 10, where the higher the score, the healthier a person is deemed to be.

### Statistical Analyses

Characteristics of participants by different HAS groups were compared using one-way analysis of variance for continuous variables and Chi-square tests for categorical variables.

The normal approximation to the Poisson distribution was used for calculating the mortality with 95% confidence intervals (CIs; Rothman & Boice, 1979). Cox regression model was used to estimate hazard ratios (HRs) and 95% CIs of death related to IC score, ES score, CD score, and HAS. The median difference (95% CI) of survival time in relation to HAS was estimated using Laplace regression. Receiver-operating characteristic (ROC) curve and the area under the curve (AUC) analyses were conducted to assess the predictive ability of three dimensions (including IC, ES, and CD) and HAS for death. DeLong’s test was used to compare two AUCs (DeLong et al., 1988). Potential additive interactions of HAS with demographic or lifestyle characteristics on the study outcome were examined based on the relative excess risk due to interaction (RERI), the attributable proportion due to interaction (AP), and the synergy index (SI; Hosmer & Lemeshow, 1992). The formulas are as follows: RERI = HR_11_ − HR_01_ − HR_10_ + 1, AP = RERI/HR_11_, and SI = (HR_11_ − 1)/[(HR_01_ − 1) + (HR_10_ − 1)], where HR_11_ indicates the HR for death associated with having both exposures, HR_01_ or HR_10_ indicates the HR for death associated with one of the exposures alone. When the 95% CI of RERI or AP does not contain 0 or S does not contain 1, there is a significantly additive interaction. The models were basic-adjusted for age, sex, and education, and further multiadjusted for marital status, current residence, annual per capita household expenditure, alcohol consumption, smoking status, and BMI.

In supplementary analyses, the association between the HAS and incident falls or hospitalization was assessed. And the sensitivity analyses were performed by using multiple imputation of chained equation (Markov chain Monte Carlo) to impute missing values on sex (*n* = 3), education (*n* = 2), and BMI (*n* = 748). The level of statistical significance was set at a *p* value less than .05. All statistical analyses were performed using Stata/SE 16.0 for Windows (StataCorp, College Station, TX) and R software version 4.1.2 (The R Foundation, Vienna, Austria).

## Results

### Characteristics of the Study Population at Baseline

Among the 5,409 participants, the mean age was 67.74 ± 6.45 years and 48% were females. Participants in the high HAS group were younger, more likely to be male, more educated, married, living in urban, with high annual per capita household expenditures, drinkers, smokers, and had lower BMI (Table 1). In addition, we have compared the characteristics between participants and dropouts (Supplementary Table 4). Compared to the participants, the dropouts were older, more likely to be female, less educated, single, with poor annual per capita household expenditure, nondrinkers, nonsmokers, and had lower BMI and higher mortality.

**Table 1.** Baseline Characteristics of the Study Population by Tertiles of the Healthy Aging Score^a^ (*n* = 5,409)

Characteristics	Poor HAS (*n* = 1,856)	Moderate HAS (*n* = 1,787)	High HAS (*n* = 1,766)	*p* Value
Age, years	69.52 ± 7.12	67.53 ± 6.19	66.08 ± 5.41	<.001
60–69	1,015 (54.69)	1,187 (66.42)	1,350 (76.44)	<.001
≥70	841 (45.31)	600 (33.58)	416 (23.56)	
Sex				<.001
Male	760 (40.97)	952 (53.30)	1,075 (60.91)	
Female	1,095 (59.03)	834 (46.70)	690 (39.09)	
Education				<.001
Illiterate	882 (47.52)	583 (32.66)	338 (19.14)	
Junior high school and below	745 (40.14)	849 (47.56)	874 (49.49)	
Senior high school and above	229 (12.34)	353 (19.78)	554 (31.37)	
Marital status				<.001
Married	1,386 (74.68)	1,435 (80.30)	1,530 (86.64)	
Single	470 (25.32)	352 (19.70)	236 (13.36)	
Current residence				<.001
Rural	1,213 (65.36)	1,109 (62.06)	904 (51.19)	
Urban	643 (34.64)	678 (37.94)	862 (48.81)	
Annual per capita household expenditure	3,396 ± 4,100	3,881 ± 4,109	4,247 ± 4,497	<.001
Low	728 (39.22)	593 (33.18)	484 (27.41)	<.001
Moderate	618 (33.30)	599 (33.52)	587 (33.24)	
High	510 (27.48)	595 (33.30)	695 (39.35)	
Alcohol consumption				<.001
No	1,456 (78.45)	1,193 (67.76)	1,084 (61.38)	
Yes	400 (21.55)	594 (33.24)	682 (38.62)	
Smoking status				<.001
Never smoking	1,141 (61.48)	988(55.29)	925 (52.38)	
Former smoking	258 (13.90)	223 (12.48)	188 (10.65)	
Current smoking	457 (24.62)	576 (32.23)	653 (36.97)	
Body mass index, kg/m^2^	23.31 ± 4.32	22.94 ± 3.90	22.76 ± 3.68	<.001
Underweight	161 (10.13)	164 (10.60)	118 (7.74)	<.001
Normal	798 (50.22)	836 (54.04)	905 (59.34)	
Overweight	427 (26.87)	395 (25.53)	410 (26.89)	
Obese	203 (12.78)	152 (9.83)	92 (6.03)	

*Notes*: Values are presented as mean ± standard deviations, or number (%). Missing data: sex = 3; education = 2; BMI = 748. BMI = body mass index; HAS = healthy aging score.

^a^The HAS was a *T*-score with a mean of 50 and a standard deviation of 10. HAS tertiles: poor group (9.54–47.17); moderate group (47.18–56.22); high group (56.23–65.60).

### Construction of HAS and Its Relationship With All-Cause Mortality

During the 7-year follow-up, 877 (16%) participants died (baseline characteristics in Supplementary Table 5). The mortality per 1,000 person-years was 26.12 (95% CI: 24.39–27.85). The relationships (univariable analysis) between each item in the three dimensions and the risk of all-cause mortality are shown in Supplementary Tables 6–8. Multivariable Cox regression analyses identified the following independent predictors for mortality: cognition (four items), mobility (seven items), and IADL domains (five items) in the IC dimension; housing domain (three items) in the ES dimension; and hypertension, diabetes, chronic lung disease, stroke, and cancer in the CD dimension (Supplementary Tables 8–10).

Likewise, each dimension score was calculated by summing corresponding domains, and the relationships of three dimensions with death were assessed. The results of the multiadjusted Cox regression model showed that the IC score (HR = 0.90, 95% CI: 0.88–0.92), ES score (HR = 0.91, 95% CI: 0.82–1.00), and CD score (reverse; HR = 0.72, 95% CI: 0.66–0.79), as continuous variables, were associated with death in a dose-dependent fashion. Compared with the high IC score group, the HRs (95% CI) of death were 1.42 (1.11–1.82) for the moderate group and 2.21 (1.72–2.84) for the poor group. Moreover, compared to participants without CD, those with one disease (HR = 1.57, 95% CI: 1.34–1.84) or with two and more diseases (HR = 2.16, 95% CI: 1.73–2.69) had a higher risk of death (Table 2).

**Table 2.** The Mortality Rate Per 1,000 Person-Years, HRs, and 95% CIs of All-Cause Mortality Related to IC Score, ES Score, and the Number of CD at Baseline

Score^a^	Mortality (95% CI)	HR (95% CI)^b^	HR (95% CI)^c^
IC score	25.40 (23.57–27.23)	0.88 (0.86–0.90)	0.90 (0.88–0.92)
Categorical IC score			
High	13.50 (10.71–16.29)	Reference	Reference
Moderate	22.16 (19.59–24.73)	1.47 (1.18–1.84)	1.42 (1.11–1.82)
Poor	38.06 (34.15–41.97)	2.50 (2.00–3.13)	2.21 (1.72–2.84)
ES score	25.40 (23.57–27.23)	0.85 (0.78–0.92)	0.91 (0.82–1.00)^d^
Categorical ES score			
High	21.09 (17.90–24.28)	Reference	Reference
Moderate	23.88 (21.29–26.48)	1.01 (0.85–1.20)	0.89 (0.73–1.82)
Poor	32.66 (28.59–36.73)	1.37 (1.14–1.64)	1.16 (0.93–1.44)
CD score (reverse)	25.40 (23.57–27.23)	0.65 (0.59–0.71)	0.72 (0.66–0.79)
Categorical CD score			
0	18.80 (16.69–20.91)	Reference	Reference
1	31.09 (27.66–34.51)	1.50 (1.30–1.74)	1.57 (1.34–1.84)
≥2	42.80 (35.14–50.45)	2.37 (1.96–2.86)	2.16 (1.73–2.69)

*Notes*: Missing data: sex = 3; education = 2; BMI = 748. BMI = body mass index; CD = chronic disease; CI = confidence interval; ES = environmental support; HR = hazard ratio; IC = intrinsic capacity.

^a^IC score categories (tertiles): poor group (0–11), moderate group (12–14), high group (15–16); ES score categories: poor group (0–1), moderate group (2), high group (3); CD indicates the number of chronic diseases, including hypertension, diabetes, chronic lung disease, stroke, and cancer; CD score ranged 0–5.

^b^Adjusted for age, sex, and education.

^c^Further adjusted for marital status, current residence, annual per capita household expenditure, alcohol consumption, smoking status, BMI, as well as IC score, ES score, and CD score, if applicable.

^d^ *p* = .047.

Further, an HAS was constructed by the equation of 3 × [ln(0.90) × IC + ln(0.91) × ES + ln(0.72) × CD]/[ln(0.90) + ln(0.91) + ln(0.72)]. The HAS (as a continuous variable) was associated with death in a dose-dependent fashion in both basic (HR = 0.955, 95% CI: 0.949–0.961) and multiadjusted (HR = 0.956, 95% CI: 0.949–0.962) Cox regression models. Compared to participants with high HAS, the risks of death were increased by 26% (HR = 1.26, 95% CI: 1.01–1.56) among those with moderate HAS and by 138% (HR = 2.38, 95% CI: 1.94–2.91) among those with poor HAS after multivariable adjustment (Table 3).

**Table 3.** The Mortality Rate Per 1,000 Person-Years, HRs with 95% CIs and 50th PDs in Survival Years in Relation to HAS: Findings from Cox Model and Laplace Repression

HAS^a^	Mortality (95% CI)	Cox Regression Model		Laplace Regression	
		HR (95% CI)^b^	HR (95% CI)^c^	50th PDs (95% CI)^b^	50th PDs (95% CI)^c^
Continuous	25.40 (23.57–27.23)	0.955 (0.949–0.961)	0.956 (0.949–0.962)	0.13 (0.11–0.15)	0.13 (0.11–0.16)
Categorical					
High	14.80 (12.56–17.04)	Reference	Reference	Reference	Reference
Moderate	21.50 (18.81–24.19)	1.24 (1.02–1.52)	1.26 (1.01–1.56)	−0.56 (−1.14 to 0.02)	−0.55 (−1.16 to 0.06)
Poor	42.77 (38.88–46.66)	2.45 (2.04–2.95)	2.38 (1.94–2.91)	−2.61 (−3.26 to −1.96)	−2.46 (−3.04 to −1.88)

*Notes*: Missing data: sex = 3; education = 2; BMI = 748. BMI = body mass index; CD = chronic disease; CI = confidence interval; ES = environmental support; HAS = healthy aging score; HR = hazard ratio; PD = percentile difference.

^a^The continuous HAS was a *T*-score with a mean of 50 and a standard deviation of 10. Categorical HAS (tertiles): poor group (9.54– 47.17); moderate group (47.18–56.22); high group (56.23– 65.60).

^b^Adjusted for age, sex, and education.

^c^Further adjusted for marital status, current residence, per capital expenditure; alcohol consumption, smoking status, and BMI.

The results of Laplace regression analyses show that the multiadjusted median time of death was 9.65 years for participants with poor HAS, 11.55 years for those with moderate HAS, and 12.11 years for those with high HAS (Supplementary Figure 2). The HAS (as a continuous variable) was associated with death in a dose-dependent fashion in both basic (50th percentile difference [PDs] = 0.13, 95% CI: 0.11–0.15) and multiadjusted (50th PDs = 0.13, 95% CI: 0.11–0.16) Laplace regression models. The 50th PDs (95% CI) of death for the participants with poor HAS were 2.46 (1.88–3.04) years earlier than those with high HAS after multiadjusted (Table 3).

**Figure 1.**
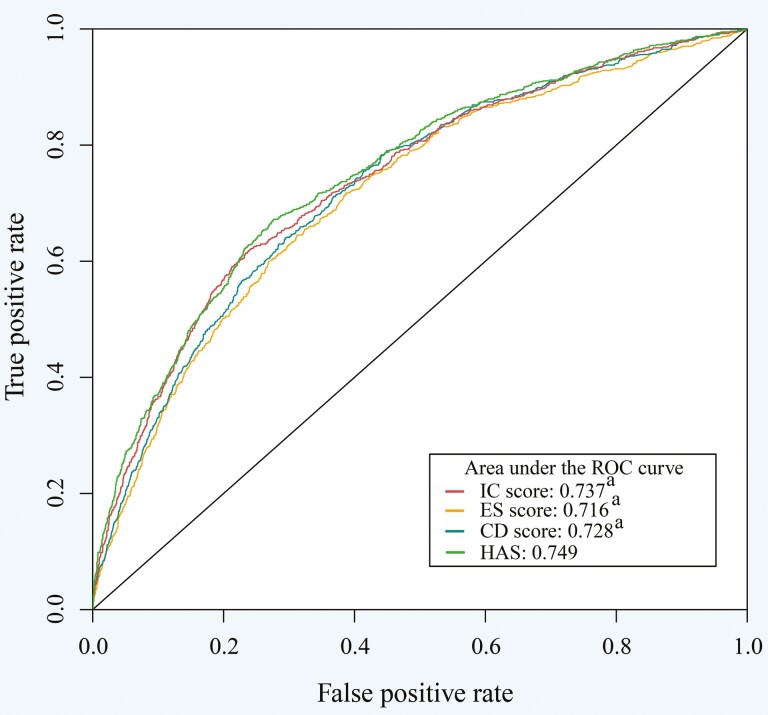
Comparison of areas under the ROC curve (AUC) for IC score, ES score, CD score (reverse scoring), and HAS. BMI = body mass index; CD = chronic disease; ES = environmental support; HAS = healthy aging score; IC = intrinsic capacity; ROC = receiver-operating characteristic. . All scores were standardized. Adjusted for age, sex, education, marital status, current residence, annual per capita household expenditure, alcohol consumption, smoking status, and BMI. ^a^Compared to HAS, *p* < .05.

In the ROC analysis, the AUCs and 95% CIs for the IC score, ES score, CD score, and HAS model were 0.737 (0.717–0.757), 0.716 (0.696–0.737), 0.728 (0.708–0.748), and 0.749 (0.729–0.768), respectively. The HAS model indicated the best prediction ability of death among the four models (*p* < .05; Figure 1 and Supplementary Table 11).

### Interactions of HAS With Demographic and Lifestyle Characteristics

**Figure 2.**
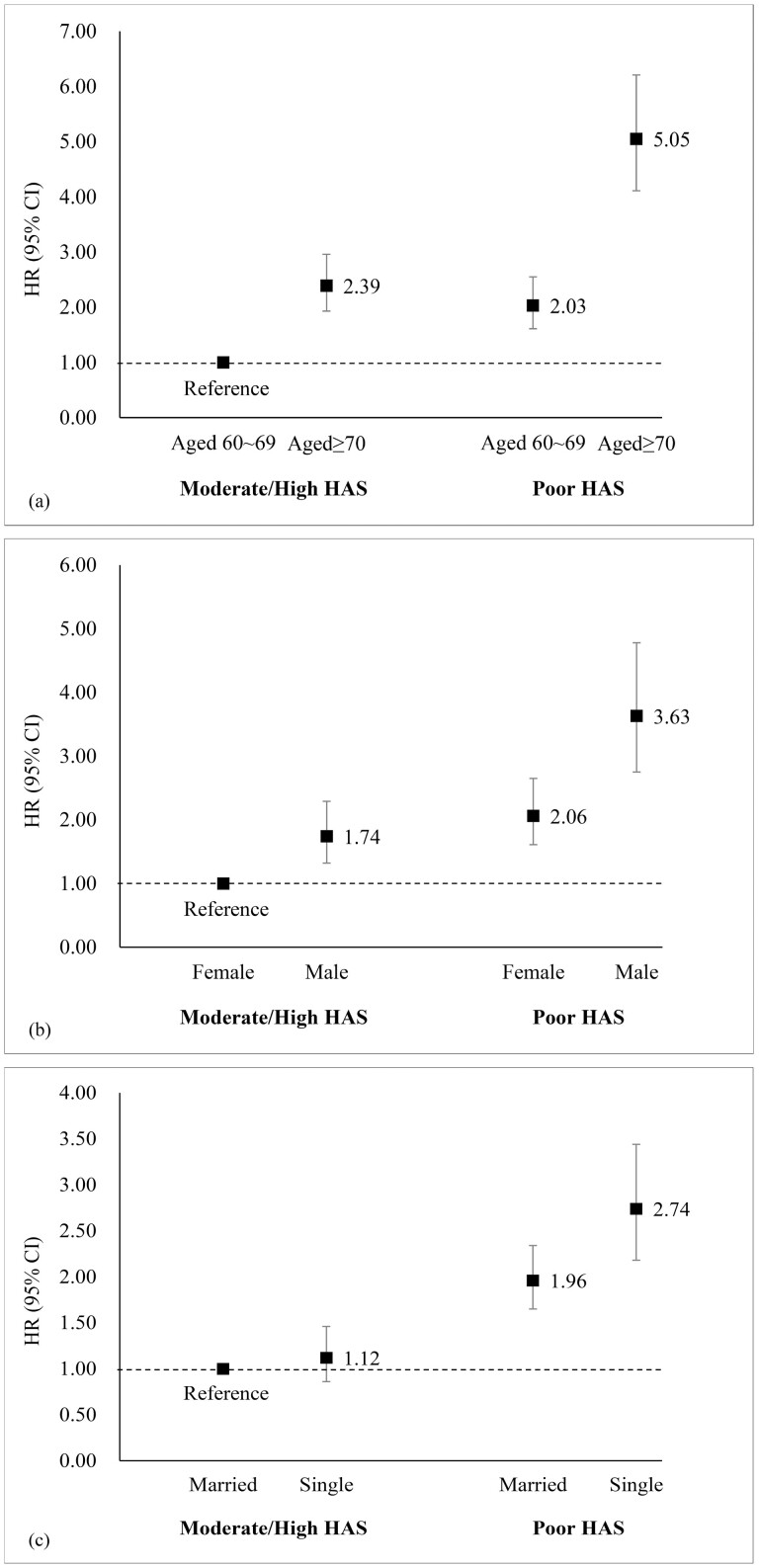
Additive interaction between HAS and age, sex, and marital status for the risk of all-cause mortality: (A) age and HAS; (B) sex and HAS; (C) marital status and HAS. Categorical HAS (tertiles): poor group (9.54–47.17); moderate/high group (47.18–65.60). Adjusted for age, sex, education, marital status, current residence, annual per capita household expenditure, alcohol consumption, smoking status, and BMI, if applicable. BMI = body mass index; CI = confidence interval; HAS = healthy aging score; HR = hazard ratio.

The multivariable Cox regression analyses were repeated for the combinations of HAS categories and different demographic and lifestyle factors (Supplementary Table 12) and the results showed that there were significant additive interactions of HAS with age, sex, and marital status in relation to death. Copresence of both poor HAS and aged ≥70 years greatly increased the HR (95% CI) for death up to 5.05 (95% CI: 4.11–6.21; Figure 2A), with significant additive interaction (RERI [95% CI]: 1.64 [0.81–2.46]; AP [95% CI]: 0.32 [0.19–0.46]; S [95% CI]: 1.68 [1.28–2.20]). Similarly, the copresence of both poor HAS and male increased the HR (95% CI) up to 3.63 (95% CI: 2.75–4.78) for death (Figure 2B), with significant additive interaction (RERI [95% CI]: 0.82 [0.16–1.49]; AP [95% CI]: 0.23 [0.07–0.39]; S [95% CI]: 1.46 [1.06–2.00]). Besides, compared to the participants who were in moderate/high HAS and married groups, the risks of death were increased by 174% (HR = 2.74, 95% CI: 2.18–3.44) among those with poor HAS and single (Figure 2C), with significant additive interaction (RERI [95% CI]: 0.65 [0.04–1.26]; AP [95% CI]: 0.24 [0.05–0.43]; S [95% CI]: 1.60 [1.02–2.52]). There were no significant interactions of HAS with education, current residence, annual per capita household expenditure, alcohol consumption, smoking status, or BMI on death.

### Supplementary Analysis

We assessed the association of HAS with incident falls or hospitalization during follow-up. Multiadjusted Cox regression showed that each point increase of HAS was associated with a lower risk of falls and hospitalization, HRs (95% CIs) were 0.979 (0.973–0.985) and 0.972 (0.966–0.977), respectively. Compared to the high HAS group, the risks were increased by 41% (HR = 1.41, 95% CI: 1.22–1.62) for falls and by 81% (HR = 1.81, 95% CI: 1.58–2.08) for hospitalization among poor HAS group (Supplementary Tables 13 and 14).

The multivariable-adjusted Cox regression for the association between HAS and mortality was reconstructed in the complete data set after imputation for missing covariates and the results did not alter much compared to those from the initial analysis (Supplementary Table 15).

## Discussion

In this national cohort study among older adults aged ≥60 years, we found that (a) cognition, mobility, and IADL domains in the IC dimension, housing domain in the ES dimension, as well as hypertension, diabetes, chronic lung disease, stroke, and cancer in the CD dimension were related to death. (b) HAS composed of IC and ES and CD was an optimal predictor of all-cause mortality, and poor HAS was related to increased all-cause mortality and premature death by more than 2 years. (c) There were significant interactions among older, male, single, and poor HAS on death.

The development of measurement for health status across populations and over time has long been a focus in the study of aging (Caballero et al., 2017). In the past decade, exploring the relationship between healthy aging and mortality in older adults has provoked widespread interest. However, the measures and operational definitions for healthy aging were inconsistent. Many studies assessed the IC dimension by integrating the clinical biomarker (e.g., systolic blood pressure, forced vital capacity, and fasting glucose) and consistently indicated that a poor IC score was associated with death (Dieteren et al., 2020; O’Connell et al., 2019; Zhang et al., 2021). Some studies have constructed IC scores including physical function (Camozzato et al., 2014; de la Fuente et al., 2018), mobility (Locquet et al., 2022), cognition function (Daskalopoulou et al., 2019), sensory (Gao et al., 2022), and psychology (Prince et al., 2021). As a result, the IC scores were reported to be a good predictor of death (Daskalopoulou et al., 2019), and a high level of IC score was related to a lower risk of death (Camozzato et al., 2014; Daskalopoulou et al., 2019; de la Fuente et al., 2018; Gao et al., 2022; Locquet et al., 2022; Prince et al., 2021). Studies on the ES dimension were mostly limited to the social component such as social networks (Santini et al., 2015), social support (Chen et al., 2021), and self-perceived support (Holt-Lunstad et al., 2010), which suggested that the poor social factors were risk factors for mortality. Moreover, older people have one or more diseases commonly, which is highly related to unhealthy aging and mortality (Boyd & Fortin, 2010; Omran, 2005). Similarly, in our study, the higher IC scores and higher ES scores were dose-dependently associated with a lower risk of death, and the absence of diseases was related to decrease mortality.

Considering that healthy aging is a multidimensional concept, several studies have assessed healthy aging from the combinations of different dimensions. A few cohort studies (Kim et al., 2019, 2021; Menec, 2003) have revealed that older people who achieved health, including the IC dimension (freedom from disability, high physical and cognitive function), ES dimension (active social engagement), and absence of major disease, might presage lower all-cause mortality. However, the dimensions in these studies were not integrated into one healthy aging indicator. Another two cohort studies (Jaspers et al., 2017; Manierre, 2019) constructed an HAS encompassing IC dimension (mental health, physical and cognitive function), ES dimension (social support and engagement), and CD, with a result that higher HAS was related to lower risk of mortality. The findings of those studies are in agreement with our results, but few studies have assessed the association between items within dimensions of healthy aging and death. However, combining a number of unscreened items into one indicator would lead to multicollinearity. Following the construction strategy of the “risk score,” we constructed a practical HAS covering multidimensional but relevant factors to maximize the prediction of outcomes. As a result, we found that cognition, mobility, and IADL domains in the IC dimension, housing domain in the ES dimension, as well as hypertension, diabetes, chronic lung disease, stroke, and cancer in the CD dimension were good predictors for death. The AUC of the HAS was larger than each dimension of HAS alone with statistically significant, which may illustrate the importance of including all health domains. In addition, the predictive power of the HAS for mortality was comparable to other studies (AUC ranged from 0.673 to 0.780; Daskalopoulou et al., 2019; Sanders et al., 2014; Swindell et al., 2010). Moreover, we found that poor HAS was associated with a higher risk of death and shortened survival. To further evaluate HAS, we assessed the predictivity of HAS for other age-related outcomes and found that poor HAS was associated with an increased risk of falls or hospitalization.

We found that there was an additive interaction between HAS and age on death. The presence of both poor HAS and being relatively older (aged ≥70 years vs 60–69 years) substantially increased mortality. Although increasing age is a risk factor for death due to function decline, older adults with poor health might not have sufficient physiological reserves to resist the intrinsic decaying effects of aging (Rowe & Kahn, 1987), leading to a fast progression to death. Our findings suggested the importance of maintaining good health in younger old age. Besides, additive interactions were observed between HAS and sex on mortality. Older male adults with poor HAS may have higher mortality. Interestingly, we also found that male participants had higher HAS compared to females (51.60 vs 48.30, *p* < .001), as in another study (Jaspers et al., 2017). This may be related to a health-survival paradox, which describes that females live longer than males but the extended life expectancy is unhealthy (Case & Paxson, 2005). Several explanations have been proposed: (a) the favorable effects of estrogen on serum lipids (Waldron & Johnston, 1976), (b) the compensatory effect of the second X chromosome (Austad, 2006), and (c) accompanied by more risk-taking behavior (Galdas et al., 2005) and more severe forms of diseases among males (Case & Paxson, 2005). As a result, unhealthy states may widen this gender gap and further increase the risk of death. Moreover, marital status seemed to be a potential effect modifier for the HAS–death association. In many studies, marital status is also a well-known predictor of health, and previous studies have shown that married people have longer survival and lower mortality (Kaplan & Kronick, 2006; Tani et al., 2018). The explanation for this is that older adults who are separated, divorced, or widowed might experience psychological and emotional stress and suffer from the loss of family support. This may have pervasive and perpetuating effects on health, increasing social vulnerability, depression, loneliness, and even social isolation (Kojima et al., 2020). It could be possible that single older adults might have more unhealthy behaviors, that is, smoking and excessive drinking compared to those with partners (Keenan et al., 2017), resulting in an elevated mortality.

The mechanisms of observed associations were complex and may have several explanations. In general, the decline in cognitive (Qiu et al., 2019) and physical (McPhee et al., 2016) functionalities are associated with accelerated aging, which further leads to impairment of IADL (Stickel et al., 2020) and mobility limitations (Schrack et al., 2013). Once these domains above of older adults can be developed and maintained, they may achieve healthy aging (WHO, 2015). People with higher cognition may be more capable of living a healthy lifestyle (Kochan et al., 2017). Besides, maintenance of cognitive function is likely to reduce the death rate by preventing the progression of dementia (Ojagbemi et al., 2016). Taken together, IC involving physical and psychosocial domains is considered as a residual biological reserve of the individual to resist age-related decline in physiological above mentioned (Cesari et al., 2018), thus reducing the risk of death. A review (Garin et al., 2014) has reported that a comfortable living environment was related to good physical and mental health, which may improve the life quality in later life and contribute to longevity. Furthermore, due to the protracted course of CD, self-management is a widely used strategy that can improve health status specifically in physical function (Jonker et al., 2009) and chronic pain (Reid et al., 2008). All in all, high HAS with strong IC, enough ES, and well-controlled CD may help postpone the onset of death in late life.

A notable strength of the study is the high-quality, nationally representative prospective cohort study with a relatively large sample. Furthermore, a comprehensive HAS was constructed based on weights of three dimensions of healthy aging including IC, ES, and CD, which incorporated the proposals from some operational definitions and the existing theoretical framework of healthy aging and addressed the knowledge gap. A novel aspect of the present study is the inclusion of potential interactions between HAS and other demographic and lifestyle factors. Nevertheless, our study involved some limitations. First, some confounding factors such as physical activity and nutrition status were not available. Second, information bias may be inevitable due to self-reported disease status. Third, the exact death date was unclear, but we assessed it by using the midpoint of two follow-up visits to minimize survival time errors. Finally, caution is required when generalizing our findings to other populations due to our findings derived from a relatively healthier population.

In conclusion, our study provides evidence that a low level of HAS encompassing IC, ES, and CD dimension is related to increased all-cause mortality and shortens survival. Our findings highlight the importance of maintaining the IC, improving the ES, and well-controlling CDs to live longer with healthy aging, especially among older, male, and single adults. Further studies are warranted to focus on the dynamic change of healthy aging over time, within individuals and between populations, and establish age- or sex-specific strategies.

## Supplementary Material

igad006_suppl_Supplementary_Material
