# Supplementary material for: Assessing Healthy Aging Score and Its Association With All-Cause Mortality: Findings From the China Health and Retirement Longitudinal Study
Source: Innov Aging. 2023 Jan 30;7(2):igad006. doi: 10.1093/geroni/igad006 (PMC10024481; doi:10.1093/geroni/igad006)
Supplement: igad006_suppl_Supplementary_Material [file igad006_suppl_supplementary_material.docx]

*Innovation in Aging* Online Supplementary Material. Zihang Zeng, Xuerui Li, Wenzhe Yang, Jiao Wang, Yun Zhu, Xiuying Qi^1^, & Weili Xu. Assessing healthy aging score and its association with all-cause mortality: findings from the China Health and Retirement Longitudinal Study.

**Supplementary Table 1.** Items and assignment of the **Intrinsic Capacity** scale in the study.

| **Intrinsic capacity** | | | | **Assignment** |
| --- | --- | --- | --- | --- |
| **Cognition** | Memory | Self-reported ratings of memory at the time of the interview. | Label | Self-comment of memory |
|  |  |  | Values | 1 = Excellent; 2 = Very good; 3 = Good; 4 = Fair; 5 = Poor |
|  |  |  | Harmonization | 1-3 into Absence; 4-5 into Presence |
|  | Immediate recall | Immediate recall of common nouns from a list. | Label | Immediate recall of 10 words |
|  |  |  | Values | Continuous variables |
|  |  |  | Harmonization | <=25% into Presence  >25% into Absence |
|  | Delayed recall | Test that assesses delayed recall using the common nouns from the list previously employed for measuring Immediate recall. | Label | Delayed recall of 10 words |
|  |  |  | Values | Continuous variables |
|  |  |  | Harmonization | <=25% into Presence  >25% into Absence |
|  | Orientation in time | Difficulties for orientation in time, are evaluated by a set of questions about the date and day of the week. | Label | Today's date: Year / Month/ Day / Day of week |
|  |  |  | Values | 1 = Year is correct; 2 = Month is correct; 3 = Day is correct  1 = Day of week correct; 2 = Day of week incorrect |
|  |  |  | Harmonization | All correct answers into Absence  Some incorrect into Presence |
|  | Numeracy | Test that assesses numeracy. | Label | What does 100 minus 7 equal? And continue minus 7 for 4 repetitions |
|  |  |  | Values | Continuous |
|  |  |  | Harmonization | All correct answers into Absence  Some incorrect into Presence |
| **Psychological symptoms** | Sleeping | Sleeping problems | Label | Sleep was restless |
|  |  |  | Values | 1 = Rarely or none of the time <1 day  2 = Some or a little of the time 1-2 days  3 = Occasionally or a moderate amount of the time 3-4 days  4 = Most or all of the time 5-7 days |
|  |  |  | Harmonization | 1 into Absence; 2-4 into Presence |
| **Vitality** | Pain | It measures if the participant experiences some degree of pain or if the participant does not present any pain at all. | Label | Currently feel any body pains |
|  |  |  | Values | 1 = yes; 2 = no |
|  |  |  | Harmonization | 2 into Absence; 1 into Presence |
|  | Energy | Self-reported high level of energy experienced at the time of the interview. | Label | Felt everything I did was an effort |
|  |  |  | Values | 1 = Rarely or none of the time <1 day  2 = Some or a little of the time 1-2 days  3 = Occasionally or a moderate amount of the time 3-4 days  4 = Most or all of the time 5-7 days |
|  |  |  | Harmonization | 1 into Absence; 2-4 into Presence |
|  | Urine incontinence | It measures if the participant has experienced loss of urine (or has used any special device for urine leakage) | Label | Have any difficulty with controlling urination and defecation? |
|  |  |  | Values | 1 = No, I don’t have any difficulty  2 = I have difficulty but can still do it  3 = Yes, I have difficulty and need help  4 = I can not do it |
|  |  |  | Harmonization | 1 into Absence; 2-4 into Presence |
| **Sensory** | Eyesight | Difficulties in eye sight using glasses or corrective lens as usual. | Label | Vision problem |
|  |  |  | Values | 1 = yes; 2 = no |
|  |  |  | Harmonization | 2 into Absence; 1 into Presence |
|  | Hearing in general | It measures if the participant experiences some difficulty for hearing (i.e., hearing someone talking on the other side of the room in a normal voice) or not, using a hearing aid as usual. | Label | Hearing problem |
|  |  |  | Values | 1 = yes; 2 = no |
|  |  |  | Harmonization | 2 into Absence; 1 into Presence |
| **Mobility** | Stooping, kneeling or crouching | Difficulty for stooping, kneeling or crouching | Label | Have difficulty stooping, kneeling, or crouching? |
|  |  |  | Values | 1 = No, I don’t have any difficulty  2 = I have difficulty but can still do it  3 = Yes, I have difficulty and need help  4 = I can not do it |
|  |  |  | Harmonization | 1 into Absence; 2-4 into Presence |
|  | Lifting or carrying weights | Difficulty for lifting or carrying weights | Label | Have difficulty lifting or carrying weights over 10 Jin? |
|  |  |  | Values | 1 = No, I don’t have any difficulty  2 = I have difficulty but can still do it  3 = Yes, I have difficulty and need help  4 = I can not do it |
|  |  |  | Harmonization | 1 into Absence; 2-4 into Presence |
|  | Climbing stairs | Difficulty for climbing stairs | Label | Have difficulty climbing several flights of stairs without resting? |
|  |  |  | Values | 1 = No, I don’t have any difficulty  2 = I have difficulty but can still do it  3 = Yes, I have difficulty and need help  4 = I can not do it |
|  |  |  | Harmonization | 1 into Absence; 2-4 into Presence |
|  | Getting up | Difficulty for getting up from sitting down | Label | Have difficulty getting up from a chair after sitting for long periods? |
|  |  |  | Values | 1 = No, I don’t have any difficulty  2 = I have difficulty but can still do it  3 = Yes, I have difficulty and need help  4 = I can not do it |
|  |  |  | Harmonization | 1 into Absence; 2-4 into Presence |
|  | Walking | Difficulty for walking by yourself and without using any special equipment | Label | - Have difficulty walking 100 meters  - Have difficulty walking 1 km |
|  |  |  | Values | 1 = No, I don’t have any difficulty  2 = I have difficulty but can still do it  3 = Yes, I have difficulty and need help  4 = I can not do it |
|  |  |  | Harmonization | Average of the two answers:  1 into Absence; 2-4 into Presence |
|  | Sitting | Difficulty for sitting for long periods | Label | Difficulty with sitting? |
|  |  |  | Values | 1 = No, I don’t have any difficulty  2 = I have difficulty but can still do it  3 = Yes, I have difficulty and need help  4 = I can not do it |
|  |  |  | Harmonization | 1 into Absence; 2-4 into Presence |
|  | Reaching or extending arms | Difficulty for reaching / extending arms | Label | Have difficulty reaching or extending your arms above shoulder level? |
|  |  |  | Values | 1 = No, I don’t have any difficulty  2 = I have difficulty but can still do it  3 = Yes, I have difficulty and need help  4 = I can not do it |
|  |  |  | Harmonization | 1 into Absence; 2-4 into Presence |
|  | Picking up | Difficulty for picking up things with fingers, e.g. Picking up a coin. | Label | Have difficulty picking up a small coin from a table? |
|  |  |  | Values | 1 = No, I don’t have any difficulty  2 = I have difficulty but can still do it  3 = Yes, I have difficulty and need help  4 = I can not do it |
|  |  |  | Harmonization | 1 into Absence; 2-4 into Presence |
| **Activities of Daily living** | Getting in or out of bed | Difficulty for getting in or out of bed | Label | Have any difficulty with getting out of bed and walking across a room? |
|  |  |  | Values | 1 = No, I don’t have any difficulty  2 = I have difficulty but can still do it  3 = Yes, I have difficulty and need help  4 = I can not do it |
|  |  |  | Harmonization | 1 into Absence; 2-4 into Presence |
|  | Bathing or showering | Difficulties for bathing or showering | Label | Have any difficulty with bathing or showering? |
|  |  |  | Values | 1 = No, I don’t have any difficulty  2 = I have difficulty but can still do it  3 = Yes, I have difficulty and need help  4 = I can not do it |
|  |  |  | Harmonization | 1 into Absence; 2-4 into Presence |
|  | Getting dressed | Difficulty for getting dressed | Label | Have any difficulty with dressing because of health and memory problem? |
|  |  |  | Values | 1 = No, I don’t have any difficulty  2 = I have difficulty but can still do it  3 = Yes, I have difficulty and need help  4 = I can not do it |
|  |  |  | Harmonization | 1 into Absence; 2-4 into Presence |
|  | Toilet | Difficulties for using the toilet | Label | Difficulty with using the toilet |
|  |  |  | Values | 1 = No, I don’t have any difficulty  2 = I have difficulty but can still do it  3 = Yes, I have difficulty and need help  4 = I can not do it |
|  |  |  | Harmonization | 1 into Absence; 2-4 into Presence |
|  | Eating | Difficulties for eating | Label | Have any difficulty with eating because of health and memory problem? |
|  |  |  | Values | 1 = No, I don’t have any difficulty  2 = I have difficulty but can still do it  3 = Yes, I have difficulty and need help  4 = I can not do it |
|  |  |  | Harmonization | 1 into Absence; 2-4 into Presence |
| **Instrumental Activities of daily living** | Housework | Difficulties for doing housework | Label | Have any difficulty with doing household chores? |
|  |  |  | Values | 1 = No, I don’t have any difficulty  2 = I have difficulty but can still do it  3 = Yes, I have difficulty and need help  4 = I can not do it |
|  |  |  | Harmonization | 1 into Absence; 2-4 into Presence |
|  | Shopping | Difficulties for shopping groceries | Label | Have any difficulty with shopping because of health and memory problem? |
|  |  |  | Values | 1 = No, I don’t have any difficulty  2 = I have difficulty but can still do it  3 = Yes, I have difficulty and need help  4 = I can not do it |
|  |  |  | Harmonization | 1 into Absence; 2-4 into Presence |
|  | Meals | Difficulties in preparing meals | Label | Have any difficulty with preparing hot meals |
|  |  |  | Values | 1 = No, I don’t have any difficulty  2 = I have difficulty but can still do it  3 = Yes, I have difficulty and need help  4 = I can not do it |
|  |  |  | Harmonization | 1 into Absence; 2-4 into Presence |
|  | Money | Difficulties for managing money, bills, or expenses | Label | Have any difficulty with managing assets because of health and memory problem? |
|  |  |  | Values | 1 = No, I don’t have any difficulty  2 = I have difficulty but can still do it  3 = Yes, I have difficulty and need help  4 = I can not do it |
|  |  |  | Harmonization | 1 into Absence; 2-4 into Presence |
|  | Medications | Difficulties for taking medications | Label | Have any difficulty with taking medications because of health and memory problem? |
|  |  |  | Values | 1 = No, I don’t have any difficulty  2 = I have difficulty but can still do it  3 = Yes, I have difficulty and need help  4 = I can not do it |
|  |  |  | Harmonization | 1 into Absence; 2-4 into Presence |

**Supplementary Table 2.** Items and assignment of the **Environmental Support** scale in the study.

| **Environmental support** | | | | **Assignment** |
| --- | --- | --- | --- | --- |
|  | | | |  |
| **Family relationships** | Visiting children | Frequency of visiting children | Label | How often do you see children? |
|  |  |  | Values | 1 = Almost every day  2 = 2-3 times a week  3 = Once a week  4 = Every two weeks  5 = Once a month  6 = Once every three months  7 = Once every six months  8 = Once a year  9 = Almost never |
|  |  |  | Harmonization | 1-5 into Absence; 6-9 into Presence |
|  | Contact with children | Frequency of contact with children | Label | How often do you have contact with children either by phone, text message, mail, or email, when you didn’t live with children? |
|  |  |  | Values | 1 = Almost every day  2 = 2-3 times a week  3 = Once a week  4 = Every two weeks  5 = Once a month  6 = Once every three months  7 = Once every six months  8 = Once a year  9 = Almost never |
|  |  |  | Harmonization | 1-5 into Absence; 6-9 into Presence |
| **Residence environment** | Residence type | Type of structure of residence | Label | What type of structure is this building? |
|  |  |  | Values | 1 = Concrete and steel  2 = Bricks and wood  3 = Mixed structure  4 = Wood, bamboo, grass  5 = Woolen felt  6 = Sheet iron  7 = Cave dwelling  8 = Tent  9 = Adobe  10 = Other |
|  |  |  | Harmonization | 1-3 into Absence; 4-10 into Presence |
|  | Kitchen | Residence with kitchen | Label | Does your residence have kitchen? |
|  |  |  | Values | 1 = Yes  2 = No |
|  |  |  | Harmonization | 1 into Absence; 2 into Presence |
|  | Toilets | Residence with toilets flushable | Label | Is the toilet flushable? |
|  |  |  | Values | 1 = Yes  2 = No |
|  |  |  | Harmonization | 1 into Absence; 2 into Presence |
|  | Electricity | Residence with electricity | Label | Does your residence have electricity |
|  |  |  | Values | 1 = Yes  2 = No |
|  |  |  | Harmonization | 1 into Absence; 2 into Presence |
|  | Water | Residence with running water | Label | Does your residence have running water? |
|  |  |  | Values | 1 = Yes  2 = No |
|  |  |  | Harmonization | 1 into Absence; 2 into Presence |
|  | Shower or bath facility | Residence with shower or bath facility | Label | Is there in-house shower or bath facility? What type |
|  |  |  | Values | 1 = Hot water provided  2 = Water heater installed by the household  3 = No |
|  |  |  | Harmonization | 1-2 into Absence; 3 into Presence |
|  | Cleanliness | Interviewer assesses the cleanliness of the residence | Label | How clear and tidy is it in this household？ |
|  |  |  | Values | 1 = Excellent  2 = Very clear  3 = Clear  4 = Fair  5 = Poor |
|  |  |  | Harmonization | 1-3 into Absence; 4-5 into Presence |
| **Community support** | Road | Road type in village/community | Label | What type of road does your village/community mainly have? |
|  |  |  | Values | 1 = Paved road  2 = Pathway/Dirt/unpaved road  3 = Sand-stone road  4 = Highway  5 = Other |
|  |  |  | Harmonization | 1 into Absence; 2-5 into Presence |
|  | Sewer system | The sewer system in the village/community | Label | Is there any sewer system in this village/community? |
|  |  |  | Values | 1 = Yes  2 = No |
|  |  |  | Harmonization | 1 into Absence; 2 into Presence |
|  | Entertainment facilities | Entertainment facilities in village/community | Label | Does your village /community have the following type of facilities? |
|  |  |  | Values | 1 = Basket-ball  2 = Swimming pool  3 = Outside exercise facilities  4 = Table tennis  5 = Room for card games and chess games  6 = Room for Ping Pong  7 = Association for calligraphy and painting  8 = Dancing team or other exercise organizations  9 = Organizations for helping the elderly and the handicapped  10 = Employment service  11 = Activity center for the elderly  12 = Elderly association  13 = Nursing Home  14 = Other entertainment facilities  15 = No |
|  |  |  | Harmonization | 1-14 into Absence; 15 into Presence |
| **Social support** | Medical insurance | Participating in insurance | Label | Are you the policy holder/primary beneficiary of any of the types of health insurance listed below? (circle all that apply) |
|  |  |  | Values | 1 = Urban employee medical insurance (yi-bao)  2 = Urban resident medical insurance  3 = New cooperative medical insurance (he-zuo-yi-liao)  4 = Urban and rural resident medical insurance  5 = Government medical insurance (gong-fei)  6 = Medical aid  7 = Private medical Insurance: Purchased by R’s union  8 = Private medical Insurance: Purchased by Individual  9 = Urban non-employed person’s health insurance  10 = Other medical insurance (specify)  11 = No insurance |
|  |  |  | Harmonization | 1-10 into Absence; 11 into Presence |
| **Social activity** | Participate in social activity | Social activity in the last month | Label | Have you done any of these activities in the last month? |
|  |  |  | Values | 1 = Interacted with friends  2 = Played Ma-jong, played chess, played cards, or went to a community club  3 = Went to a sport, social, or other kinds of club  4 = Took part in a community-related organization  5 = Done voluntary or charity work  6 = Attended an educational or training course  7 = None of these |
|  |  |  | Harmonization | 1-6 into Absence; 7 into Presence |

**Supplementary Table 3.** Items and assignment of the **Chronic Disease** in the study.

| Have you been diagnosed with these diseases by a doctor? No or Yes |
| --- |
| (1) Hypertension |
| (2) Dyslipidemia (elevation of low density lipoprotein, triglycerides (TGs), and total cholesterol, or a low high density lipoprotein level) |
| (3) Diabetes or high blood sugar |
| (4) Cancer or malignant tumor (excluding minor skin cancers) |
| (5) Chronic lung diseases, such as chronic bronchitis, emphysema (excluding tumors, or cancer) |
| (6) Liver disease (except fatty liver, tumors, and cancer) |
| (7) Heart attack, coronary heart disease, angina, congestive heart failure, or other heart problems |
| (8) Stroke |
| (9) Kidney disease (except for tumor or cancer) |
| (10) Stomach or other digestive diseases (except for tumor or cancer) |
| (11) Emotional, nervous, or psychiatric problems |
| (12) Memory-related disease |
| (13) Arthritis or rheumatism |
| (14) Asthma |

**Supplementary Table 4**. Baseline characteristics and survival status between dropouts and participants.

| **Characteristics** | Dropouts (n=2,272) | Participants (n=5,409) | *P* value |
| --- | --- | --- | --- |
| **Age, years** | 70.28 ± 8.12 | 67.74 ± 6.45 | <0.001 |
| 60~69 | 1,193 (52.51) | 3,552 (65.67) | <0.001 |
| ≥70 | 1,079 (47.49) | 1,857 (34.33) |  |
| **Sex** |  |  | <0.001 |
| Male | 1,058 (46.61) | 2,787 (51.55) |  |
| Female | 1,212 (53.39) | 2,619 (48.45) |  |
| **Education** |  |  | <0.001 |
| Illiterate | 1,035 (45.86) | 1,803 (33.35) |  |
| Junior high school and below | 886 (39.26) | 2,468 (45.64) |  |
| Senior high school and above | 336 (14.89) | 1,136 (21.01) |  |
| **Marital status** |  |  | <0.001 |
| Married | 1,624 (71.64) | 4,351 (80.44) |  |
| Single | 643 (28.36) | 1,058 (19.56) |  |
| **Current residence** |  |  | 0.084 |
| Rural | 1,400 (61.76) | 3,226 (59.64) |  |
| Urban | 867 (38.24) | 2,183 (40.36) |  |
| **Annual per capita household expenditure** | 2333 (1048, 4263) | 2683 (1425, 4880) | <0.001 |
| Low | 880 (38.73) | 1,684 (31.13) | <0.001 |
| Moderate | 724 (31.87) | 1,834 (33.91) |  |
| High | 668 (29.40) | 1,891 (34.96) |  |
| **Alcohol consumption** |  |  | <0.001 |
| No | 1,615 (73.11) | 3,733 (69.01) |  |
| Yes | 594 (26.89) | 1,676 (30.99) |  |
| **Smoking status** |  |  | <0.001 |
| Never smoking | 1,358 (68.10) | 3,054 (56.46) |  |
| Former smoking | 175 (8.78) | 669 (12.37) |  |
| Current smoking | 461 (23.12) | 1,686 (31.17) |  |
| **Body mass index, kg/m^2^** | 22.42 ± 4.00 | 23.01 ± 3.99 | <0.001 |
| Underweight | 198 (13.16) | 443 (9.29) | <0.001 |
| Normal | 753 (50.07) | 2,539 (53.25) |  |
| Overweight | 313 (20.81) | 1,232 (25.84) |  |
| Obese | 240 (15.96) | 554 (11.62) |  |
| **Survival status** |  |  | <0.001 |
| Survival | 1,436 (71.16) | 4,532 (83.79) |  |
| Died | 582 (28.84) | 877 (16.21) |  |
| Note. Missing data: sex=5; education=17; marital status=5; current residence=5; alcohol consumption=63; smoking status=278; BMI=1639; lost follow-up=254. | | | |

| **Supplementary Table 5**. Baseline characteristics of the study population by survival status (n =5,409). | | | |
| --- | --- | --- | --- |
| **Characteristics** | **Death** | **Survival** | ***P* value** |
|  | **(n=877)** | **(n=4,532)** |  |
| **Age, years** | 71.79 ± 7.32 | 66.95 ± 5.95 | <0.001 |
| 60~69 | 357 (40.71) | 3,195 (70.50) | <0.001 |
| ≥70 | 520 (59.29) | 1,337 (29.50) |  |
| **Sex** |  |  | <0.001 |
| Male | 545 (62.21) | 2,242 (49.49) |  |
| Female | 331 (37.79) | 2,288 (50.51) |  |
| **Education** |  |  | <0.001 |
| Illiterate | 353 (40.25) | 1,450 (31.01) |  |
| Junior high school and below | 396 (45.15) | 2,072 (45.74) |  |
| Senior high school and above | 128 (14.60) | 1,008 (22.25) |  |
| **Marital status** |  |  | <0.001 |
| Married | 646 (73.66) | 3,705 (81.75) |  |
| Single | 231 (26.34) | 827 (18.25) |  |
| **Current residence** |  |  | 0.013 |
| Rural | 556 (63.40) | 2,670 (58.81) |  |
| Urban | 321 (36.60) | 1,862 (41.19) |  |
| **Annual per capita household expenditure** | 3,373 ± 3,527 | 3,923 ± 4,371 | <0.001 |
| Low | 357 (40.71) | 1,448 (31.95) | <0.001 |
| Moderate | 277 (31.58) | 1,527 (33.69) |  |
| High | 243 (27.71) | 1,557 (34.36) |  |
| **Alcohol consumption** |  |  | 0.920 |
| No | 604 (68.87) | 3,129 (69.04) |  |
| Yes | 273 (31.13) | 1,403 (30.96) |  |
| **Smoking status** |  |  | <0.001 |
| Never smoking | 401 (45.72) | 2,653 (58.54) |  |
| Former smoking | 171 (19.50) | 498 (10.99) |  |
| Current smoking | 305 (34.78) | 1,381 (30.47) |  |
| **Body mass index, kg/m^2^** | 22.16 ± 3.83 | 23.17 ± 4.00 | <0.001 |
| Underweight | 111 (15.00) | 332 (8.47) | <0.001 |
| Normal | 414 (55.95) | 2,125 (54.20) |  |
| Overweight | 162 (21.98) | 1,070 (27.29) |  |
| Obese | 53 (7.16) | 394 (10.05) |  |
| **Intrinsic capacity score** ^a^ | 1.62 ± 0.70 | 1.94 ± 0.75 | <0.001 |
| Poor | 441 (50.29) | 1,403 (30.96) |  |
| Moderate | 325 (37.06) | 1,985 (43.80) |  |
| High | 111 (12.65) | 1,144 (25.24) |  |
| **Environmental support score** ^b^ | 1.90 ± 0.75 | 2.06 ± 0.74 | <0.001 |
| Poor | 295 (33.64) | 1,112 (24.54) |  |
| Moderate | 374 (42.65) | 2,017 (44.51) |  |
| High | 208 (23.71) | 1,403 (30.95) |  |
| **Chronic diseases score ^c^** | 0.69 ± 0.70 | 0.48 ± 0.60 | <0.001 |
| 0 | 390 (44.47) | 2,620 (57.81) | <0.001 |
| 1 | 367 (41.85) | 1,660 (36.63) |  |
| ≥2 | 120 (13.68) | 252 (5.56) |  |
| Note. Values are presented as mean ± standard deviations, or number (%).  ^a^ Intrinsic capacity score categories (tertiles): low group (0 to 11), moderate group (12 to 14), high group (15 to 16);  ^b^ Environmental support score categories (tertiles): low group (0 to 1), moderate group (2), high group (3).  ^c^ Chronic disease includes hypertension, diabetes, chronic lung disease, stroke, and cancer; The ranged was 0~5.  Missing data: sex=3; education=2; BMI=748. | | | |

| **Supplementary Table 6**. Intrinsic capacity domains and items of the study population by survival status (n =5,409). | | | |
| --- | --- | --- | --- |
| **Intrinsic capacity domain and item** | **Death** | **Survival** | ***P* value** |
|  | **(n=877)** | **(n=4,532)** |  |
| **Cognition** |  |  |  |
| Memory | 158 (18.02) | 842 (18.58) | 0.694 |
| Orientation in time | 278 (31.70) | 1,828 (40.34) | <0.001 |
| Numeracy | 244 (27.80) | 1,713 (37.82) | <0.001 |
| Immediate recall | 566 (64.54) | 3,484 (76.88) | <0.001 |
| Delayed recall | 525 (59.86) | 3,298 (72.77) | <0.001 |
| **Psychological symptoms** |  |  |  |
| Sleeping | 421 (48.00) | 2,172 (47.93) | 0.955 |
| **Vitality** |  |  |  |
| Pain | 569 (64.88) | 3,025 (66.76) | 0.280 |
| Energy | 343 (39.11) | 2,124 (46.87) | <0.001 |
| Urine incontinence | 797 (90.88) | 4,319 (95.30) | <0.001 |
| **Sensory** |  |  |  |
| Near vision | 262 (29.87) | 1,434 (31.64) | 0.302 |
| Far vision | 238 (27.14) | 1,496 (33.01) | 0.001 |
| Eyesight | 768 (87.57) | 4,210 (92.89) | <0.001 |
| Hearing in general | 300 (34.21) | 1,795 (39.61) | 0.003 |
| **Mobility** |  |  |  |
| Stooping, kneeling or crouching | 483 (55.07) | 3,008 (66.37) | <0.001 |
| Lifting or carrying weights | 641 (73.09) | 3,933 (86.78) | <0.001 |
| Climbing stairs | 327 (37.29) | 2,462 (54.32) | <0.001 |
| Getting up | 519 (59.18) | 3,144 (69.37) | <0.001 |
| Walking | 632 (72.06) | 4,137 (91.28) | <0.001 |
| Reaching or extending arms | 720 (82.10) | 4,063 (89.65) | <0.001 |
| Picking up | 797 (90.88) | 4,370 (96.43) | <0.001 |
| **ADL** |  |  |  |
| Getting in or out of bed | 776 (88.48) | 4,272 (94.26) | <0.001 |
| Bathing or showering | 713 (81.30) | 4,260 (94.30) | <0.001 |
| Getting dressed | 777 (88.60) | 4,304 (94.97) | <0.001 |
| Toilet | 673 (76.74) | 3,878 (85.57) | <0.001 |
| Eating | 819 (93.39) | 4,410 (97.31) | <0.001 |
| **IADL** |  |  |  |
| Housework | 680 (77.54) | 4,079 (90.00) | <0.001 |
| Shopping | 697 (79.48) | 4,119 (90.89) | <0.001 |
| Meals | 675 (76.97) | 4,132 (91.17) | <0.001 |
| Money | 666 (75.94) | 3,966 (87.51) | <0.001 |
| Medications | 777 (88.60) | 4,234 (93.42) | <0.001 |
| Note. Values are presented as number (%).  Abbreviations: IC, intrinsic capacity. | | | |

| **Supplementary Table 7**. Environmental support domains and items of the study population by survival status (n =5,409). | | | |
| --- | --- | --- | --- |
| **Environmental support domain and item** | **Death** | **Survival** | ***P* value** |
|  | **(n=877)** | **(n=4,532)** |  |
| **Outdoor spaces and buildings** |  |  |  |
| Road | 590 (67.27) | 3,076 (67.87) | 0.729 |
| Public toilet | 340 (38.77) | 1,729 (38.15) | 0.730 |
| Outdoor exercise places | 473 (53.93) | 2,520 (55.60) | 0.362 |
| Indoor entertainment venues | 386 (44.01) | 2,226 (49.12) | 0.006 |
| **Housing** |  |  |  |
| House type | 712 (81.19) | 3,875 (85.50) | 0.001 |
| Handicapped facilities | 215 (24.52) | 1,082 (23.87) | 0.684 |
| Electricity supply | 668 (76.17) | 3,768 (83.14) | <0.001 |
| Water supply | 515 (58.72) | 2,824 (62.31) | 0.045 |
| Shower facilities | 238 (27.14) | 1,558 (34.38) | <0.001 |
| **Communications and information** |  |  |  |
| Phone | 381 (43.44) | 2,364 (52.16) | <0.001 |
| Internet | 81 (9.24) | 521 (11.50) | 0.051 |
| **Community support** |  |  |  |
| Institutions or venues for the older people | 458 (52.22) | 2,475 (54.61) | 0.194 |
| Unemployment subsidies | 80 (9.12) | 575 (12.69) | 0.003 |
| Minimum living allowance | 747 (85.18) | 3,760 (82.97) | 0.108 |
| **Transportation** | 514 (58.61) | 2,849 (62.86) | 0.017 |
| **Social participation** | 376 (42.87) | 2,200 (48.54) | 0.002 |
| Note. Values are presented as number (%).  Abbreviations: ES, environmental support. | | | |

| **Supplementary Table 8.** The mortality rate per 1,000 person-years, hazard ratios (HRs), and 95% confidence intervals (CIs) of all-cause mortality related to chronic diseases at baseline. | | | |
| --- | --- | --- | --- |
| **Disease** | **Mortality (95%CI)** | **Univariable analysis ^a^** | **Multivariable analysis ^b^** |
| Dyslipidemia | 21.35 (16.65 to 26.06) | 0.81 (0.64 to 1.02) | - |
| Arthritis | 25.52 (22.68 to 28.35) | 0.96 (0.84 to 1.10) | - |
| Digest disease | 26.05 (22.29 to 29.82) | 1.00 (0.85 to 1.18) | - |
| **Hypertension** | 26.12 (24.39 to 27.85) | 1.28 (1.12 to 1.47) | **1.27 (1.09 to 1.49)** |
| Heart disease | 32.24 (27.41 to 37.08) | 1.29 (1.10 to 1.53) | 1.19 (0.98 to 1.45) |
| Kidney disease | 35.17 (26.75 to 43.59) | 1.37 (1.07 to 1.76) | 1.16 (0.88 to 1.54) |
| **Diabetes** | 36.69 (29.06 to 44.31) | 1.45 (1.16 to 1.80) | **1.33 (1.03 to 1.73)** |
| Liver disease | 37.44 (24.86 to 50.03) | 1.45 (1.03 to 2.04) | 1.41 (0.95 to 2.09) |
| Psychiatric problems | 39.05 (18.60 to 59.51) | 1.48 (0.87 to 2.50) | - |
| Asthma | 39.79 (31.28 to 48.30) | 1.57 (1.25 to 1.96) | 0.94 (0.72 to 1.24) |
| **Chronic lung disease** | 45.14 (38.79 to 51.49) | 1.92 (1.64 to 2.26) | **1.46 (1.19 to 1.78)** |
| Memory-related disease | 50.62 (35.49 to 65.75) | 1.96 (1.44 to 2.66) | 1.12 (0.77 to 1.62) |
| **Stroke** | 61.27 (46.60 to 75.94) | 2.45 (1.91 to 3.14) | **1.87 (1.38 to 2.53)** |
| **Cancer** | 78.84 (43.39 to 11.43) | 3.03 (1.92 to 4.77) | **2.76 (1.68 to 4.52)** |
| ^a^ Unadjusted model.  ^b^ Adjusted for age, sex, education, marital status, current residence, annual per capita household expenditure, alcohol consumption, smoking status, BMI, as well as other diseases, if applicable. | | | |

| **Supplementary Table 9.** The hazard ratios (HRs), and 95% confidence intervals (CIs) of all-cause mortality related to IC domains at baseline. | | |
| --- | --- | --- |
| **Intrinsic capacity domain** | **HR (95%CI) ^a^** | **HR (95%CI) ^b^** |
| **Cognition** | **0.90 (0.84–0.95)** | **0.92 (0.86–0.98)** |
| Vitality | 0.98 (0.87–1.11) | 1.03 (0.90–1.18) |
| Sensory | 1.00 (0.92–1.09) | 1.03 (0.94–1.12) |
| **Mobility** | **0.86 (0.81–0.90)** | **0.86 (0.81–0.90)** |
| ADL | 1.04 (0.96–1.13) | 1.05 (0.96–1.15) |
| **IADL** | **0.89 (0.83–0.95)** | **0.88 (0.82–0.94)** |
| Note. Abbreviations: IC, intrinsic capacity; ADL, activity of daily living; IADL, instrumental activity of daily living.  ^a^ Adjusted for age, sex, and education.  ^b^ Further adjusted for marital status, current residence, annual per capita household expenditure, alcohol consumption, smoking status, BMI, as well as cognition, vitality, sensory, mobility, ADL, and IADL, if applicable. | | |

| **Supplementary Table 10.** The hazard ratios (HRs), and 95% confidence intervals (CIs) of all-cause mortality related to ES domains at baseline. | | |
| --- | --- | --- |
| **Environmental support domain** | **HR (95%CI) ^a^** | **HR (95%CI) ^b^** |
| Outdoor spaces and buildings | 0.95 (0.83–1.04) | 0.89 (0.76–1.04) |
| **Housing** | **0.89 (0.80–0.98)** | **0.89 (0.81–0.99)** |
| Communications and information | 0.91 (0.78–1.05) | 0.88 (0.76–1.03) |
| Community support | 0.94 (0.71–1.23) | 0.93 (0.70–1.23) |
| Transportation | 0.95 (0.83–1.10) | 0.97 (0.83–1.14) |
| Social participation | 0.88 (0.76–1.02) | 0.91 (0.79–1.06) |
| Note. Abbreviations: ES, environmental support.  ^a^ Adjusted for age, sex, education.  ^b^ Further adjusted for marital status, current residence, annual per capita household expenditure, alcohol consumption, smoking status, BMI, as well as outdoor spaces and buildings, housing, communications and information, community support, transportation, and social participation, if applicable. | | |

| **Supplementary Table 11.** Comparison of different area under the ROC curve (AUC). | | | | | |
| --- | --- | --- | --- | --- | --- |
| **Model** | **AUC** | **95%CI** | ***P* ^a^** | ***P* ^b^** | ***P* ^c^** |
| IC | 0.737 | 0.717 to 0.757 | Reference |  |  |
| ES | 0.716 | 0.696 to 0.737 | <0.001 | Reference |  |
| CD | 0.728 | 0.708 to 0.748 | 0.095 | 0.009 | Reference |
| HAS | 0.749 | 0.729 to 0.768 | <0.001 | <0.001 | <0.001 |
| Note. IC, intrinsic capacity; ES, environmental support; CD, chronic diseases; HAS, heathy aging score.  ^a^ Compared to IC.  ^b^ Compared to ES.  ^c^ Compared to CD. | | | | | |

**Supplementary Table 12.** Additive interactions between HAS and demographic characteristics.

| **Group** | **Mortality (95%CI)** | **HR (95%CI) ^a^** | **RERI**  **(95%CI)** | **AP**  **(95%CI)** | **S**  **(95%CI)** |
| --- | --- | --- | --- | --- | --- |
| **HAS & age** |  |  |  |  |  |
| Moderate/High HAS & aged 60~69 | 12.21 (10.41 to 14.02) | Reference | **1.64**  **(0.81 to 2.46)** | **0.32**  **(0.19 to 0.46)** | **1.68**  **(1.28 to 2.20)** |
| Moderate/High HAS & aged ≥70 | 34.04 (29.05 to 39.02) | **2.39 (1.93 to 2.96)** |  |  |  |
| Poor HAS & aged 60~69 | 23.12 (19.15 to 27.05) | **2.03 (1.61 to 2.55)** |  |  |  |
| Poor HAS & aged ≥70 | 68.21 (60.67 to 75.75) | **5.05 (4.11 to 6.21)** |  |  |  |
| **HAS & sex** |  |  |  |  |  |
| Moderate/High HAS & female | 11.57 (9.29 to 13.85) | Reference | **0.82**  **(0.16 to 1.49)** | **0.23**  **(0.07 to 0.39)** | **1.46**  **(1.06 to 2.00)** |
| Moderate/High HAS & male | 22.95 (20.13 to 25.77) | **1.74 (1.32 to 2.29)** |  |  |  |
| Poor HAS & female | 29.60 (25.19 to 34.01) | **2.06 (1.61 to 2.65)** |  |  |  |
| Poor HAS & male | 58.56 (50.67 to 66.44) | **3.63 (2.75 to 4.78)** |  |  |  |
| **HAS & education** |  |  |  |  |  |
| Moderate/High HAS & Senior high school and above | 12.28 (9.07 to 15.50) | Reference | -0.62  (-1.72 to 0.49) | -0.19  (-0.54 to 0.14) | 0.78  (0.53 to 1.15) |
| Moderate/High HAS & Junior high school and below | 19.79 (17.54 to 22.03) | **1.61 (1.21 to 2.16)** |  |  |  |
| Poor HAS & Senior high school and above | 40.09 (28.25 to 51.94) | **3.16 (2.13 to 4.70)** |  |  |  |
| Poor HAS & Junior high school and below | 40.74 (36.41 to 45.06) | **3.16 (2.35 to 4.25)** |  |  |  |
| **HAS & marital status** |  |  |  |  |  |
| Moderate/High HAS & married | 17.28 (15.27 to 19.29) | Reference | **0.65**  **(0.04 to 1.26)** | **0.24**  **(0.05 to 0.43)** | **1.60**  **(1.02 to 2.52)** |
| Moderate/High HAS & single | 21.95 (16.85 to 27.06) | 1.12 (0.86 to 1.46) |  |  |  |
| Poor HAS & married | 35.67 (31.31 to 40.03) | **1.96 (1.65 to 2.34)** |  |  |  |
| Poor HAS & single | 56.55 (46.75 to 66.35) | **2.74 (2.18 to 3.44)** |  |  |  |
| **HAS & current residence** |  |  |  |  |  |
| Moderate/High HAS & urban | 17.23 (14.30 to 20.15) | Reference | -0.21  (-0.72 to 0.30) | -0.11  (-0.38 to 0.16) | 0.81  (0.51 to 1.28) |
| Moderate/High HAS & rural | 18.58 (16.13 to 21.02) | 0.94 (0.75 to 1.17) |  |  |  |
| Poor HAS & urban | 41.60 (34.34 to 48.87) | **2.17 (1.69 to 2.79)** |  |  |  |
| Poor HAS & rural | 40.22 (35.32 to 45.12) | **1.90 (1.52 to 2.37)** |  |  |  |
| **HAS & annual per capita household expenditure** |  |  |  |  |  |
| Moderate/High HAS & moderate/high | 16.35 (14.19 to 18.51) | Reference | 0.17  (-0.35 to 0.68) | 0.07  (-0.14 to 0.27) | 1.13  (0.77 to 1.67) |
| Moderate/High HAS & low | 21.73 (18.06 to 25.39) | 1.19 (0.95 to 1.47) |  |  |  |
| Poor HAS & moderate/high | 37.13 (32.12 to 42.14) | **2.09 (1.72 to 2.55)** |  |  |  |
| Poor HAS & low | 45.96 (39.13 to 52.79) | **2.45 (1.98 to 3.01)** |  |  |  |
| **HAS & alcohol consumption** |  |  |  |  |  |
| Moderate/High HAS & no | 21.10 (17.69 to 24.51) | Reference | -0.34  (-0.88 to 0.20) | -0.17  (-0.47 to 0.12) | 0.74  (0.45 to 1.20) |
| Moderate/High HAS & yes | 16.37 (14.15 to 18.60) | 1.07 (0.86 to 1.33) |  |  |  |
| Poor HAS & no | 45.44 (36.87 to 54.02) | **2.22 (1.84 to 2.66)** |  |  |  |
| Poor HAS & yes | 38.96 (34.47 to 43.45) | **1.95 (1.52 to 2.49)** |  |  |  |
| **HAS & smoking status** |  |  |  |  |  |
| Moderate/High HAS & never smoking | 13.04 (10.87 to 15.21) | Reference | 0.47  (-0.09 to 1.03) | 0.16  (-0.02 to 0.35) | 1.33  (0.92 to 1.92) |
| Moderate/High HAS & former/current smoking | 23.97 (20.77 to 27.17) | **1.36 (1.07 to 1.72)** |  |  |  |
| Poor HAS & never smoking | 30.89 (26.42 to 35.35) | **2.06 (1.64 to 2.58)** |  |  |  |
| Poor HAS & former/current smoking | 57.25 (49.33 to 65.16) | **2.89 (2.28 to 3.66)** |  |  |  |
| **HAS & body mass index** |  |  |  |  |  |
| Moderate/High HAS & overweight/obese | 20.09 (17.64 to 22.54) | Reference | -0.24  (-0.71 to 0.23) | -0.14  (-0.42 to 0.14) | 0.76  (0.45 to 1.29) |
| Moderate/High HAS & underweight/normal | 14.16 (11.33 to 17.00) | 0.88 (0.69 to 1.12) |  |  |  |
| Poor HAS & overweight/obese | 47.35 (41.66 to 53.04) | **2.11 (1.77 to 2.51)** |  |  |  |
| Poor HAS & underweight/normal | 30.91 (25.35 to 36.46) | **1.75 (1.40 to 2.19)** |  |  |  |
| Note. Abbreviations: RERI, relative excess risk due to interaction; AP, attributable proportion due to interaction; SI, synergy index. The HAS was a T-score with a mean of 50 and a standard deviation of 10. Categorical HAS (tertiles): poor group (9.54 to 47.09); moderate/high group (47.18 to 65.60). Missing data: sex=3; education=2; BMI=748.  ^a^ Adjusted for age, sex, education, marital status, current residence, annual per capita household expenditure; alcohol consumption, smoking status, and BMI, if applicable. | | | | | |

| **Supplementary Table 13.** The hazard ratios (HRs), and 95% confidence intervals (CIs) of incident falls related to healthy aging score (HAS). | | |
| --- | --- | --- |
| **Healthy aging score** | **HR (95%CI) ^a^** | **HR (95%CI) ^b^** |
| **Continuous** | 0.980 (0.975 to 0.985) | 0.979 (0.973 to 0.985) |
| **Categorical** |  |  |
| High | Reference | Reference |
| Moderate | 1.08 (0.95 to 1.23) | 1.09 (0.95 to 1.25) |
| Poor | 1.41 (1.24 to 1.61) | 1.41 (1.22 to 1.62) |
| ^a^ Adjusted for age, sex, education.  ^b^ Further adjusted for marital status, current residence, annual per capita household expenditure, alcohol consumption, smoking status, and BMI. | | |

| **Supplementary Table 14.** The hazard ratios (HRs), and 95% confidence intervals (CIs) of incident hospitalizations related to healthy aging score (HAS). | | |
| --- | --- | --- |
| **Healthy aging score** | **HR (95%CI) ^a^** | **HR (95%CI) ^b^** |
| **Continuous** | 0.975 (0.970 to 0.980) | 0.972 (0.966 to 0.977) |
| **Categorical** |  |  |
| High | Reference | Reference |
| Moderate | 1.39 (1.23 to 1.58) | 1.38 (1.21 to 1.58) |
| Poor | 1.78 (1.56 to 2.02) | 1.81 (1.58 to 2.08) |
| ^a^ Adjusted for age, sex, education.  ^b^ Further adjusted for marital status, current residence, annual per capita household expenditure, alcohol consumption, smoking status, and BMI. | | |

| **Supplementary Table 15.** The mortality rate per 1,000 person-years, hazard ratios (HRs) with 95% confidence intervals (CIs) and 50th percentile differences (PDs) in survival years in relation to healthy aging score (HAS): findings from Cox model and Laplace repression with multiple imputation for missing data on covariates. | | | | | |
| --- | --- | --- | --- | --- | --- |
| **HAS ^a^** | **Mortality (95%CI)** | **Cox Regression Model** | | **Laplace Regression** | |
|  |  | **HR (95%CI) ^b^** | **HR (95%CI) ^c^** | **50^th^ PDs (95%CI) ^b^** | **50^th^ PDs (95%CI) ^c^** |
| **Continuous** | 26.12 (24.39 to 27.85) | 0.955 (0.949 to 0.961) | 0.956 (0.950 to 0.962) | 0.13 (0.12 to 0.15) | 0.14 (0.12 to 0.16) |
| **Categorical** |  |  |  |  |  |
| High | 14.50 (12.29 to 16.70) | Reference | Reference | Reference | Reference |
| Moderate | 21.43 (18.73 to 24.12) | 1.33 (1.09 to 1.63) | 1.29 (1.05 to 1.57) | -0.57 (-1.15 to 0.01) | -0.47 (-1.04 to 0.10) |
| Poor | 43.40 (39.47 to 47.34) | 2.62 (2.17 to 3.15) | 2.51 (2.08 to 3.02) | -2.61 (-3.26 to -1.96) | -2.45 (-2.99 to -1.90) |
| ^a^ The continuous HAS was a T-score with a mean of 50 and a standard deviation of 10. Categorical HAS (tertiles): poor group (9.42 to 47.03); moderate group (47.12 to 56.12); high group (56.28 to 65.63).  ^b^ Adjusted for age, sex, and education.  ^c^ Further adjusted for marital status, current residence, per capital expenditure; alcohol consumption, smoking status, and BMI. | | | | | |


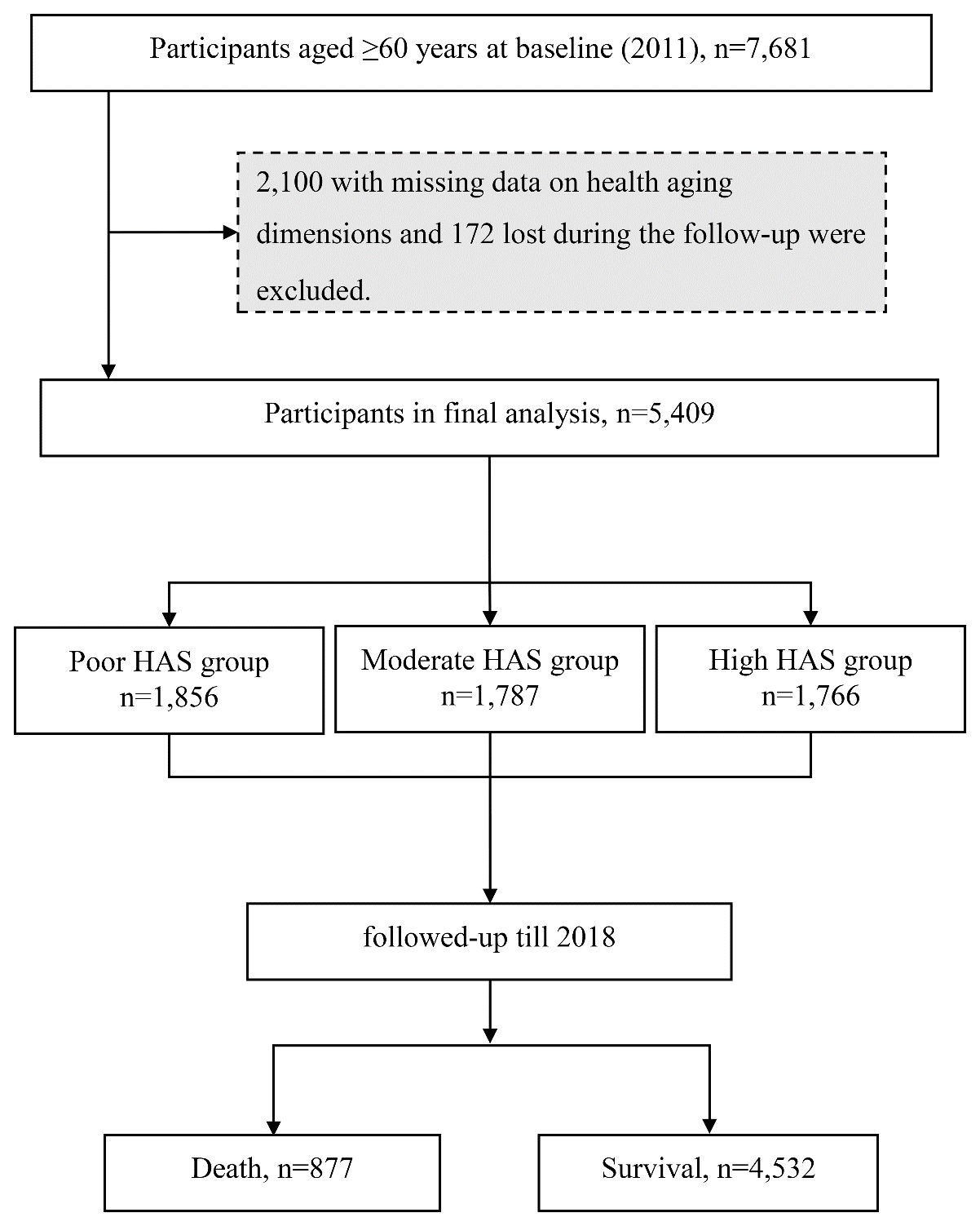


**Supplementary Figure 1.** Flowchart of study participants.

**
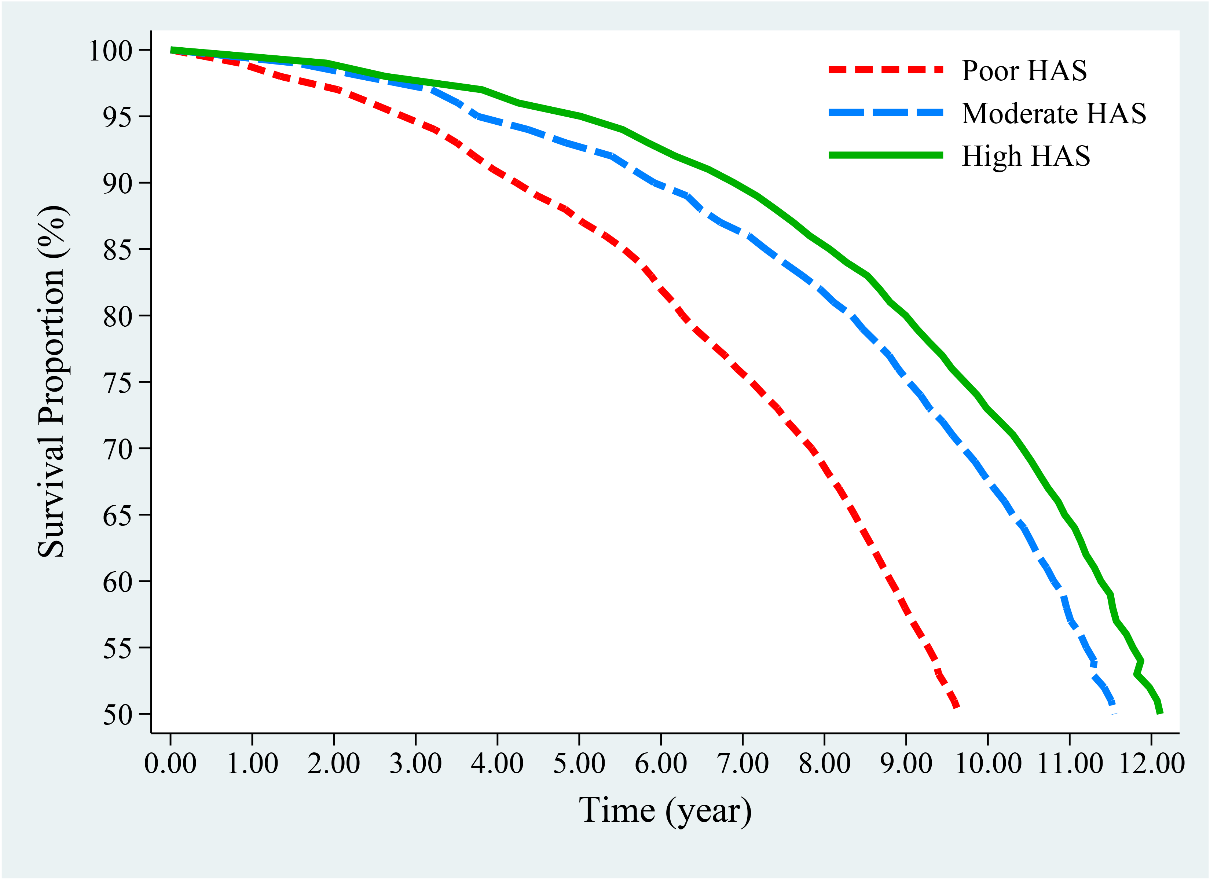
**

**Supplementary Figure 2.** Laplace regression analysis of 50th percentile differences in years of survival by Healthy Aging Score (HAS) subgroups.

The HAS was a T-score with a mean of 50 and a standard deviation of 10. Categorical HAS (tertiles): poor group (9.54 to 47.09); moderate group (47.18 to 56.08); high group (56.23 to 65.60). The model was adjusted for age, sex, education, marital status, current residence, annual per capita household expenditure, alcohol consumption, smoking status, and BMI.
